# Anejaculation as an atypical presentation of prostate cancer: a case report

**DOI:** 10.1186/1757-1626-1-81

**Published:** 2008-08-11

**Authors:** Uwais Mufti, Khurshid R Ghani, Rateb Samman, Jaspal Virdi, Bernard Potluri

**Affiliations:** 1 Department of Urology, Princess Alexandra Hospital, Hamstel Road, Harlow, Essex, CM20 1QX, UK

## Abstract

Anejaculation may occur as a result of neurological disease, iatrogenic injury or be drug induced. We report a case of a 66 year old man who presented with anejaculation following an emergency abdominal aortic aneurysm repair. Due to an elevated prostate specific antigen (PSA) level, the patient underwent a prostate biopsy and was diagnosed with a prostate adenocarcinoma. This was effectively managed using active surveillance, a treatment modality that aims to select only those patients with significant cancer for radical treatment. Despite the possible cause of anejaculation to be iatrogenic, the reader should be aware that prostate cancer may co-exist in, or cause any disorder of the lower urinary tract.

## Background

Anejaculation is defined as the complete absence of antegrade or retrograde ejaculation [1]. It is caused by failure of emission of semen from the prostate and seminal ducts into the urethra. In the older man, prostate cancer is the diagnosis of exclusion.

## Case presentation

A 66 year old man with a history of hypertension and ischaemic heart disease was referred by his general practitioner complaining of anejaculation. He had no difficulty in obtaining erections or orgasm and no history of lower urinary tract symptoms. Anejaculation coincided with recent emergency abdominal aortic aneurysm (AAA) repair. Digital rectal examination (DRE) revealed a moderately enlarged smooth prostate. The prostate specific antigen (PSA) level was 4.5 ng/l (age related normal range 0–4). The likely cause for anejaculation in this patient was injury to the sympathetic trunk during AAA repair. The differential diagnosis was retrograde ejaculation and prostate cancer. Semen analysis of the first voided urine following intercourse excluded retrograde ejaculation. After informed consent the patient underwent a 14-core transrectal ultrasound (TRUS) guided prostate biopsy. This revealed a moderately differentiated prostate adenocarcinoma (Gleason score 6) in only 2 cores (largest focus of cancer = 2 mm). Following case discussion at a specialist multi-disciplinary team meeting, the patient was offered three prostate cancer management options: radical radiotherapy, brachytherapy or active surveillance. Radical prostatectomy was not a suitable option due to cardiac risk factors. The patient opted for active surveillance and was referred to the Medical Research Council active surveillance study at the Royal Marsden Hospital, London.

## Discussion

Ejaculation is mediated by the sympathetic nervous system. Causes of anejaculation include spinal cord injury, cauda equina lesions, multiple sclerosis, Parkinson's disease, diabetes mellitus, medication (antihypertensive, antipsychotic, antidepressants, alcohol) and surgery (aortoiliac surgery, retro peritoneal lymph node dissection, colorectal resection) [1]. The thoracolumbar sympathetic nerves cause contraction of the smooth muscles of the prostate, seminal vesicles and vas deferens leading to emission of seminal fluid into the urethra. These nerves are prone to injury during AAA repair, especially when undertaken as an emergency [2]. Aortoiliac surgery can also lead to damage of the superior hypogastric plexus and result in erectile dysfunction. In one study, up to 80% of patients had some form of sexual dysfunction after AAA surgery [3]. There are no published figures on the rate of anejaculation only.

Even though a diagnosis of post-AAA repair anejaculation may have been evident in this case, sexual dysfunction is also a recognised presentation of prostate cancer. The case we have presented is the first reported case of prostate cancer detected in a patient complaining of anejaculation. When DRE finding or PSA level is unable to confidently exclude prostate cancer, patients should be advised of the risk of prostate cancer. Systematic TRUS guided prostate biopsy is the gold standard method for diagnosing prostate cancer. As a result of the PSA test, the majority of modern day prostate cancers are PSA detected cancers. These patients often have early localised asymptomatic cancers.

Active surveillance is the most recent management option in the treatment of localised prostate cancer. It aims to individualise the management of early low grade (Gleason score <= 7) prostate cancer by selecting only those men with significant cancers for curative treatment. It involves strict monitoring of the PSA level, DRE finding and histological status by repeat prostate biopsy. Early, radical treatment is offered to those with evidence of significant biochemical or histological progression [4]. This is in contrast to watchful waiting which for decades has been associated with less stringent observation criteria with late, palliative treatment offered to those who develop symptoms of cancer progression. Early results of active surveillance have been encouraging. A Royal Marsden Hospital study found that 80% of 80 patients recruited to active surveillance continued to be under surveillance after a median of 42 months follow up. 14% of patients received radical treatment of which all remained biochemically controlled with no evidence of recurrent disease. None of the patients in the study developed metastatic prostate cancer and there were no deaths from prostate cancer [5]. A more recent study has examined the effect of delaying radical treatment in active surveillance [6]. 38 active surveillance patients were compared to 150 similar patients who underwent immediate surgical intervention. The median interval for delayed surgical intervention was 26.5 months (range 12–73 months). The investigators found that delaying radical surgery did not compromise curability.

Active surveillance aims to avoid the morbidity associated with radical treatment and identify those men with clinically insignificant prostate cancer. Although mature data from studies on active surveillance is currently limited, it seems to be an important management option to consider in men with early low grade prostate cancer.

## Competing interests

The authors declare that they have no competing interests.

## Authors' contributions

UM and KRG were involved in collection of data and material used during preparation of the paper. UM drafted an initial version of the paper. KRG was involved in the inception, drafting and editing of the paper. RS, JSV and BSP contributed to the initial draft. BSP is the lead clinician involved in the care of the patient and the prime supervisor of the work. All authors read and approved the final manuscript.

## Consent

Written informed consent was obtained from the patient for publication of this case report. A copy of the written consent is available for review by the Editor-in-Chief of this journal.

## References

[B1] Colpi G, Weidner W, Jungwirth A, Pomerol J, Papp G, Hargreave T, Dohle G (2004). EAU Guidelines on Disorders of Ejaculation. European Urology.

[B2] Jimenez JC, Smith MM, Wilson SE (2004). Sexual dysfunction in men after open or endovascular repair of abdominal aortic aneurysms. Vascular.

[B3] Lee ES, Kor DJ, Kuskowski MA (2000). Incidence of erectile dysfunction after open abdominal aortic aneurysm repair. Ann Vasc Surg.

[B4] Parker C (2003). Active Surveillance: an individualised approach to early prostate cancer. BJU Int.

[B5] Hardie C, Parker C, Norman A, Eeles R, Horwich A, Huddart R, Deamaley D (2005). Early outcomes of active surveillance for localized prostate cancer. BJU Int.

[B6] Warlick Christopher, Trock BruceJ, Landis Patricia, Epstein JonathanI, Carter H Ballentine (2006). Delayed Versus Immediate Surgical Intervention and Prostate Cancer Outcome. J National Cancer Inst.

